# Clinical and genetic findings in two siblings with X-Linked agammaglobulinemia and bronchiolitis obliterans: a case report

**DOI:** 10.1186/s12887-022-03245-x

**Published:** 2022-04-05

**Authors:** Ronaldo da Silva Francisco Junior, Guilherme Loss de Morais, Joseane Biso de Carvalho, Cristina dos Santos Ferreira, Alexandra Lehmkuhl Gerber, Ana Paula de C Guimarães, Flávia Anisio Amendola, Fernanda Pinto-Mariz, Zilton Farias Meira de Vasconcelos, Ekaterini Simões Goudouris, Ana Tereza Ribeiro de Vasconcelos

**Affiliations:** 1 Bioinformatics Laboratory-LABINFO, National Laboratory of Scientific Computation LNCC/MCTIC, Av. Getulio Vargas, 333, Quitandinha CEP: 25651-075 Petrópolis, Rio de Janeiro, Brazil; 2 Laboratory of High Complexity of the Fernandes Figueira Institute (LACIFF) - Oswaldo Cruz Foundation (FIOCRUZ), Rio de Janeiro, Brazil; 3 Allergy and Immunology Service of the Martagão Gesteira Institute for Childcare and Pediatrics (IPPMG) - Federal University of Rio de Janeiro (UFRJ), Rio de Janeiro, Brazil

**Keywords:** X-linked agammaglobulinemia, *BTK*, Whole-exome sequencing, Bronchiolitis obliterans

## Abstract

**Background:**

X-linked agammaglobulinemia (XLA) is an Inborn Errors of Immunity (IEI) characterized by pan-hypogammaglobulinemia and low numbers of B lymphocytes due to mutations in *BTK* gene. Usually, XLA patients are not susceptible to respiratory tract infections by viruses and do not present interstitial lung disease (ILD) such as bronchiolitis obliterans (BO) as a consequence of acute or chronic bacterial infections of the respiratory tract. Although many pathogenic variants have already been described in XLA, the heterogeneous clinical presentations in affected patients suggest a more complex genetic landscape underlying this disorder.

**Case presentation:**

We report two pediatric cases from male siblings with X-Linked Agammaglobulinemia and bronchiolitis obliterans, a phenotype not often observed in XLA phenotype. The whole-exome sequencing (WES) analysis showed a rare hemizygous missense variant NM_000061.2(BTK):c.1751G>A(p.Gly584Glu) in *BTK* gene of both patients. We also identified a gain-of-function mutation in *TGFβ1* (rs1800471) previously associated with transforming growth factor-beta1 production, fibrotic lung disease, and graft fibrosis after lung transplantation. TGFβ1 plays a key role in the regulation of immune processes and inflammatory response associated with pulmonary impairment.

**Conclusions:**

Our report illustrates a possible role for WES in patients with known inborn errors of immunity, but uncommon clinical presentations, providing a personalized understanding of genetic basis, with possible implications in the identification of potential treatments, and prognosis for patients and their families.

**Supplementary Information:**

The online version contains supplementary material available at 10.1186/s12887-022-03245-x.

## Background

X-Linked Agammaglobulinemia (XLA, OMIM entry 300,755) is a classic model of predominantly antibody deficiencies caused by mutations in Bruton’s Tyrosine Kinase (*BTK*) gene (OMIM #300300) with a reported incidence rate of 1/100,000 or 1/200,000 live births. The cytoplasmic tyrosine kinase protein encoded by *BTK* gene plays an important role in signal transduction during B cell maturation [1, 2]. Due to arrest of B cell development at the pre-B or mature B cells differentiation stages, affected patients usually display reduced total immunoglobulin (Ig) production and peripheral B cells frequency (CD19 and CD20) less than 1–2% [1, 3]. Male patients with XLA frequently undergo recurrent infection episodes such as otitis, pneumonia, sinusitis, and arthritis during childhood.

This increased risk for pulmonary complications, even after Ig replacement therapy (IRT), is often caused by bronchiectasis and interstitial lung disease (ILD) due to bacterial infections [4, 5]. However, ILD shows a significantly variable clinical manifestation among patients with primary antibody deficiency (PAD), suggesting the influence of antibody-independent factors not yet defined. Cases of ILD in XLA patients are atypical when compared to common variable immunodeficiency (CVID), and, overall, they are reported by multicenter studies focusing on general clinical and laboratory comparison rather than genetic and epigenetic findings [6]. Genetic contributions to the progression of lung complications in XLA regardless of IRT are still not elucidated.

Here, we report two pediatric cases from male siblings with severe B lymphopenia, recurrent infection episodes, and antibody deficiency diagnosed as XLA. Interestingly, both patients presented severe pulmonary impairment diagnosed as bronchiolitis obliterans using tomographic criteria, not often observed in XLA. We carried out a comprehensive whole-exome sequencing study in the subjects aiming to identify candidate genetic variants possibly related to the both clinical presentations observed. Variant prioritization was conducted using the American College of Medical Genetics and Genomics (ACMG) and the Association for Molecular Pathology (AMP) guidelines for sequence variants analysis in order to provide a precise genetic diagnosis and better comprehend the genetic mechanisms underlying XLA in our patients.

## Case presentation

Patient 1 (P1) and 2 (P2) are two male siblings from a non-consanguineous couple, born in the state of Rio de Janeiro, Brazil. They had a past clinical history of recurrent respiratory infections, mainly pneumonia, severe wheezing, and sepsis, with symptoms onset being observed before the first year of life in P1 and at 2 y.o for P2. Severe wheezing and secondary bacterial pneumonia were part of the initial presentation. Both children were first seen during their first ICU hospitalization. The XLA diagnosis was established at 11 months and 2y5m of age in each subject, respectively. At the time this study was conducted, P1 was 3 y.o and P2 was 5 y.o.

Immunological findings revealed CD19 and CD20 less than 1% in both patients. P1 showed levels of IgG < 270 mg/dL (Reference Value for Brazilian patients (RV), RV: 520–875 mg/dL), IgM = 12 mg/dL (RV: 47–138 mg/dL), and IgA = 20 mg/dL (RV: 7–130 mg/dL). On the other hand, P2 had IgG = 100 mg/dL (RV: 540–116 mg/dL), IgM = 24.9 mg/dL (RV: 43–194 mg/dL), IgA < 5 mg/dL (RV: 11–192 mg/dL). Both had undetectable levels of IgE isotype.

Patients were kept on the regular infusion of intravenous immunoglobulin replacement and prophylaxis with azithromycin after the diagnosis. They were receiving Immunoglobulin reposition doses (600 mg/kg every 21 days IV). We kept both on azithromycin as soon as they were discharged from ICU. They had already been diagnosed as probable XLA due to agammaglobulinemia and low B cells and presenting persistent wheezing.

**Fig. 1:**
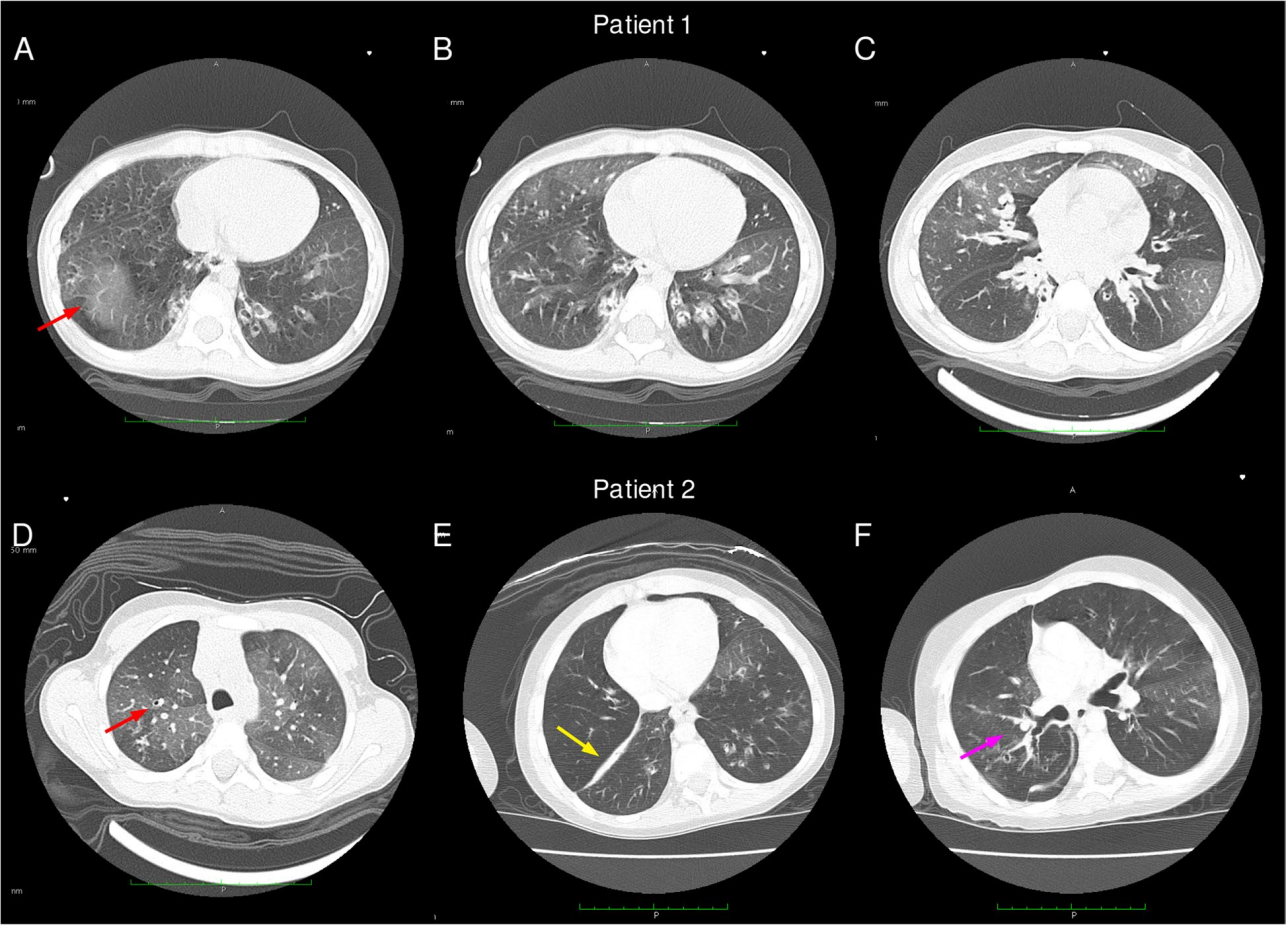
Thorax CT imaging of the two study subjects. Upper and lower rows indicate patient 1 (**a-c**) and patient 2 (**d-f**) images, respectively. **a-c**) was obtained in 2016 at 3 y.o of P1. Chest CT scan in **d**) was obtained in 2015 at 3y7mo, whereas **e** and **f** were taken in late 2017 at 6 y.o. Red arrows indicate mosaic attenuation patterns and Bronchiectasis. Yellow arrow shows atelectasis. In pink, we showed a large bronchiectasis and bronchial wall thickening

Both patients had recurrent and severe wheezing and images suggesting bronchiolitis obliterans were observed in the thorax computed tomography (CT; Figs. 1 and S1). They evolved with recurrent respiratory viral infections, severe bronchospasm, and the need for several hospitalizations, as well as worsening of CT images. In both patients, no severe bacterial infections have been observed since the beginning of immunoglobulin replacement. Currently, patient 1 is 6 y.o and has partial control of the bronchospasm, even with high doses of inhaled corticosteroids, associated with long-acting beta-agonists (LABA) and long-acting muscarinic antagonist (LAMA). Now he presents signs of chronic pulmonary disease: increase in chest anteroposterior diameter and digital clubbing. Patient 2, evolved with more severe pulmonary disease and was kept also on high doses of inhaled corticosteroid, LABA and LAMA, plus oral cyclosporine and frequent oral corticosteroid short treatments. However, he died with respiratory insufficiency at 6 y.o.

On average, whole-exome sequencing (WES) achieved 99% of the reads mapped to the human reference genome with the mean depth of coverage greater than 100X in 96% of exonic regions. A total of 73,668 SNVs were identified among common and rare variants, being 40,372 shared in both patients. Due to the potential presence of non-synonymous variants in genes previously associated with inborn errors of immunity (IEI), we queried an enriched set of genes related to the agammaglobulinemia phenotype (HP:0,004,432) according to HPO [7]. From the 19 genes filtered, we only found variants in the *BTK* gene of both patients. The variant was located in the exon 16 of the NM_000061.2 transcript, the candidate to be the principal isoform of the gene (Figure S2). The missense variant changes a nonpolar residue of Glycine at the codon 584 by a polar neutrally charged Glutamate in the TK domain of the BTK protein. NM_000061.2(BTK):c.1751G>A (p.Gly584Glu) was not found in control samples from the 1000 Genomes Project and the Genome Aggregation Database (GnomAD). This variant is located on the exonic side of the splice site with a predicted loss-of-function (pLOF) effect (gene has 121 pathogenic LOF variants and gnomAD Loss-of-Function Observed/Expected = 0 is less than 0.755). The codon 584 is located in a mutational hot-spot with 17 amino acids harboring 7 non-VUS (7 pathogenic and 0 benign). Missense variants are a common driven mechanism of XLA disease in *BTK*. We also observed 12 pathogenic predictions from BayesDel_addAF, CADD, DEOGEN2, FATHMM-MKL, LIST-S2, M-CAP, MVP, MutationAssessor, MutationTaster, Polyphen2-HVAR, PrimateAI, and SIFT versus no benign predictions. This variant was also reported as pathogenic in the literature in patients with XLA [8, 9]. Altogether, Gly584Glu was classified as Pathogenic by Varsome using the following ACMG/AMP criteria: PVS1, PM1, PM2, PP2, PP3.

Next, we retrieved the HPO terms for bronchiolitis obliterans (HP:0,011,946), bronchiolitis (HP:0,011,950), bronchospasm (HP:0,025,428) and pneumonia (HP:0,002,090) phenotypes in HPO database. An additional list of genes associated with bronchiolitis was also retrieved from the Human Gene Mutation Database (HGMD; see supplementary material). We found a missense variant (rs1800471) in the *TGFβ1* gene reported as a disease-associated polymorphism with supporting functional evidence in HGMD. The Arg25Pro has been associated with transforming growth factor-beta1 production, fibrotic lung disease, and graft fibrosis after lung transplantation.

We also predicted the classic HLA alleles [HLA-A, HLA-B, HLA-C, HLA-DP (DPA1, DPB1, and DPB2), HLA-DQ (DQA1, DQA2, and DQB1), and HLA-DR (DRA, DRB1, 3, 4, and 5)] for each patient using NGS data (Table S1). Patient 1 carried the haplotype DRB3*02:02 associated with bronchospasm [10]. Three other haplotypes DRB1*11:01, DQB1*03:01, and DQA1*05 were found in this subject previously associated with sepsis and pneumonia [11–13]. HLA alleles associated with symptoms of bronchospasm were also found in Patient 2 (DPB1*03:01), which also harbored DQB1*05:02 and DRB1*16:02 often found in patients with sepsis [14, 15].

## Discussion and conclusions

The patients reported here were diagnosed with XLA after an initial episode of probably bacterial lung infection associated with sepsis and severe bronchospasm. Since the beginning of regular replacement of intravenous immunoglobulin and initial use of prophylactic antibiotics, they have had no relevant bacterial infection. However, the clinical course of both was characterized by recurrent and severe episodes of wheezing, many of them requiring hospitalization and insufficient response to the proposed treatment. The clinical and radiological images of both patients were compatible with the diagnosis of post-infectious bronchiolitis obliterans. Post-infectious bronchiolitis obliterans occurs most commonly after viral respiratory tract infections by adenovirus, or more rarely related to bacterial infections [16]. Overall, patients with XLA are not susceptible to respiratory tract infections by viruses and also do not usually present BO as a consequence of acute or chronic bacterial infections of the respiratory tract [6, 17].

Bronchiolitis obliterans (BO) is an ILD characterized by subepithelial inflammation and airway obstruction due to fibrosis of the bronchioles, which leads to defects in epithelial and airway regeneration. BO is often associated with lung or hematopoietic stem cell transplants, autoimmune disorders, inhalation of toxic agents, and post-infectious episodes. Barnes et al. 2015 reported a series of XLA patients who developed BO after lung transplantation [18]. Adenovirus infection is among the main potential causes of BO from post-infectious origins [19, 20]. Weinberger et al. 2019 estimated a rare incidence of less than 1% of BO manifestation in XLA patients from the US Immunodeficiency Network [21]. For this reason, the mechanisms underlying the risks of developing BO in XLA are still poorly understood. Even with the use of regular IRT with proper dosage, infections on the surface of the respiratory tract may continue to occur. This constitutes an important factor for the development of bronchiectasis and chronic lung disease, which is usually proportional to the years of the disease.

In the present study, we detected a heterozygous gain-of-function (GoF) variant in *TGFβ1* (rs1800471) in two siblings with clinical manifestations of X-Linked Agammaglobulinemia. Although the rs1800471 seems to play a role in the phenotype observed in our patients, the limited number of samples does not allow us to draw conclusions about its association with the phenotype of bronchiolitis obliterans. Further studies are needed to confirm this association in other patients with IEI, particularly those with defects in antibody production, and the diagnosis of post-infectious bronchiolitis obliterans. The haplotype of classic HLA alleles may also contribute to the phenotype observed once similar related symptoms were described in the literature in other individuals. Both HLA alleles and *TGFβ1* variant seem not to have a causative effect, instead, we hypothesized that they might be associated with the phenotypes only based on the evidence available in the literature. Therefore, patients were diagnosed with XLA and may have modifiers genes. Altogether, our report illustrates a possible role for WES in patients with known IEI and uncommon clinical presentations, providing a personalized understanding of genetic basis, with possible implications in the identification of potential treatments, and prognosis for patients and their families.

## Supplementary Information

**Additional file 1: Table S1.** Prediction of HLA alleles present in each patient. **Figure S1.** Chest CT scan of patient 2. a-c) was obtained in 2015 at 3y7mo, whereas d was obtained in late 2017 at 6 y.o. Cyan arrows show bronchial wall thickening. Red arrows indicate mosaic attenuation patterns and Bronchiectasis. Yellow arrow shows atelectasis. **Figure S2.** Genetic diagnosis of XLA patients using WES. a) Ideogram of the human X chromosome, in red the region q22.1 where the *BTK* gene is located, and the *BTK* isoform taken from UCSC Genome Browser. b) Family pedigree showing the inheritance model of BTK mutation in both siblings. c) The box represents a zoom in the region where the mutation is located. Red and blue rectangles represent the forward and reverse NGS reads covering the variants, followed by the Sanger sequencing eletrofluorograms for the same region.

## Data Availability

The sequencing data used in our study are publicly available in SRA-NCBI (www.ncbi.nlm.nih.gov/sra), SRA accession: PRJNA818322.

## Abbreviations

XLA: X-linked agammaglobulinemia
IEI: Inborn errors of immunity
ILD: Interstitial lung disease
BO: Bronchiolitis obliterans
WES: Whole-exome sequencing
BTK: Bruton’s Tyrosine Kinase
PAD: Primary antibody deficiency
CVID: Common variable immunodeficiency
CD19: Cluster of Differentiation 19
CD20: Cluster of Differentiation 20
Ig: Immunoglobulin
IRT: Immunoglobulin replacement therapy
AMP: Association for Molecular Pathology
ACMG: American College of Medical Genetics and Genomics
CT: Computer tomography
LABA: Long-acting beta-agonists
LAMA: Long-acting muscarinic antagonist
