# Supplementary material for: Clinical and genetic findings in two siblings with X-Linked agammaglobulinemia and bronchiolitis obliterans: a case report
Source: BMC Pediatr. 2022 Apr 5;22:181. doi: 10.1186/s12887-022-03245-x (PMC8981605; doi:10.1186/s12887-022-03245-x)
Supplement: Supplementary file 1 — Additional file 1: Table S1. Prediction of HLA alleles present in each patient. Figure S1. Chest CT scan of patient 2. a-c) was obtained in 2015 at 3y7mo, whereas d was obtained in late 2017 at 6 y.o. Cyan arrows show bronchial wall thickening. Red arrows indicate mosaic attenuation patterns and Bronchiectasis. Yellow arrow shows atelectasis. Figure S2. Genetic diagnosis of XLA patients using WES. a) Ideogram of the human X chromosome, in red the region q22.1 where the BTK gene is located, and the BTK isoform taken from UCSC Genome Browser. b) Family pedigree showing the inheritance model of BTK mutation in both siblings. c) The box represents a zoom in the region where the mutation is located. Red and blue rectangles represent the forward and reverse NGS reads covering the variants, followed by the Sanger sequencing eletrofluorograms for the same region. [file 12887_2022_3245_MOESM1_ESM.zip › Supplementary_Material_Francisco_Jr_et_al_2021_R2.docx]

**Supplementary Material**

**Clinical and Genetic Findings in Two Siblings with X-Linked Agammaglobulinemia and Bronchiolitis Obliterans: a case report**

Ronaldo da Silva Francisco Junior^1^, Guilherme Loss de Morais^1^, Joseane Biso de Carvalho^1^, Cristina dos Santos Ferreira^1^, Alexandra Lehmkuhl Gerber^1^, Ana Paula de C Guimarães^1^, Flávia Anisio Amendola^2^, Fernanda Pinto-Mariz^3^, Zilton Farias Meira de Vasconcelos^2^ , Ekaterini Simões Goudouris^3^, and Ana Tereza Ribeiro de Vasconcelos^1,^**^*^**

**1** Bioinformatics Laboratory-LABINFO - National Laboratory of Scientific Computation LNCC/MCTIC

**2** Laboratory of High Complexity of the Fernandes Figueira Institute (LACIFF) - Oswaldo Cruz Foundation (FIOCRUZ)

**3** Allergy and Immunology Service of the Martagão Gesteira Institute for Childcare and Pediatrics (IPPMG) - Federal University of Rio de Janeiro (UFRJ)

*** Corresponding author:** Ana Tereza Ribeiro de Vasconcelos, Bioinformatics Laboratory-LABINFO, National Laboratory of Scientific Computation LNCC/MCTIC, Av. Getulio Vargas, 333, Quitandinha CEP: 25651-075 - Petrópolis - Rio de Janeiro - Brazil, Work (+5524) 2233-6065, fax (+5524) 2233-6124, e-mail: atrv@lncc.br.

**Methods**

### **Study subjects**

We studied two male siblings with a clinical history suggestive of XLA. The patients were originated from a Brazilian family in the state of Rio de Janeiro. Patients were treated at the Instituto de Puericultura e Pediatria Martagão Gesteira (IPPMG) – Universidade Federal do Rio de Janeiro (UFRJ). At the time of the study, both patients received regular intravenous immunoglobulin replacement. Both of them presented recurrent infection episodes, circulant B cells (CD19) levels < 1%, reduced levels of serum immunoglobulin production, and remarkable pulmonary impairment.

**DNA extraction and Sequencing**

Peripheral blood lymphocytes were obtained from the patients in order to isolate genomic DNA using the kit QIAmp DNA Mini Kit® (QIAGEN®) according to the manufacturer’s instructions. Next, we performed WES using the Illumina TruSeq® Exome Kit (8 rxn × 6plex) to prepare the exome libraries according to the manufacturer’s protocols. Sequencing was performed using Illumina NextSeq® 500/550 High Output Kit v2 (150 cycles), generating 2x75 bp paired-end reads. We validated the mutation found in *BTK* gene by Sanger sequencing in each patient. Using the PureLink PCR purification kit (Life Technologies, Carlsbad, CA, USA), we purified the PCR product amplified with specific primers flanked the variant regions. Next, we quantified the amplicons in the IMPLAN nanophotometer and sequenced using BigDye® Terminator v3.1 Cycle Sequencing Kit in the ABI 3130 (Applied Biosystems, Foster, CA, USA). The Sanger sequences and the Exome reads aligned were visualized using UGENE toolkit and IGV, respectively [[1]](https://paperpile.com/c/e5Wprt/Q44n).

**Bioinformatics analysis**

We processed the raw NGS data files for each patient separately. Short reads mapping to the human reference genome (GRCh38/hg38) was performed using Bowtie2 version 2.3.4.1 [[2]](https://paperpile.com/c/e5Wprt/wi8b). The output files in SAM format were converted to BAM files, sorted and filtered by MAPping Quality > 30 using samtools version 1.3 [[3]](https://paperpile.com/c/e5Wprt/PzSY). Duplicated reads were later identified using MarkDuplicates from Picard software version 2.18 (http://picard.sourceforge.net/). Next, we called Single Nucleotide Variants (SNVs) and small insertion and deletions (INDELS) by using the UnifiedGenotyper tool from Genome Analysis Toolkit (GATK) software version 3.8 [[4,5]](https://paperpile.com/c/e5Wprt/9KDO+W6rf). We followed the best practices steps for variant calling including variant quality filtration and base recalibration according to the GATK protocols [[5]](https://paperpile.com/c/e5Wprt/W6rf). SnpEff and SnpSift software version 4.3 [[6]](https://paperpile.com/c/e5Wprt/6D4n) were used to predict genetic effects and molecular impacts for the variants called. The Minor Allele Frequency (MAF) for each variant was annotated according to the variant frequencies in dbSNP [[7]](https://paperpile.com/c/e5Wprt/K3zs), 1000Genomes [[8]](https://paperpile.com/c/e5Wprt/AFwp), ExAC and GnomAD [[9]](https://paperpile.com/c/e5Wprt/xdAU). We queried an enriched set of genes previously described as associated with XLA and BO in HPO. We also searched for mutations in an addition set of genes reported in HGMD (*NOD2, TLR3, COPA, CCL5, KLRK1, TLR4, NFKBIA, TLR5, IFNAR2, NSMCE3, IL1RN, IL27*) as associated with BO. Finally, we used Varsome (<https://varsome.com/>) to annotate ACMG classification for interpretation of clinical genetic variant effects. Prediction of HLA haplotypes was performed using the HLAminer tool [[10]](https://paperpile.com/c/e5Wprt/2Wce) with the FASTQ files generated during the next-generation sequencing.

**Supplementary Tables**

**Table S1. Prediction of HLA alleles present in each patient.**

**Supplementary Figures**

**Figure S1. Chest CT scan of patient 2.** **a-c**) was obtained in 2015 at 3y7mo, whereas d was obtained in late 2017 at 6 y.o. Cyan arrows show bronchial wall thickening. Red arrows indicate mosaic attenuation patterns and Bronchiectasis. Yellow arrow shows atelectasis.

**Figure S2. Genetic diagnosis of XLA patients using WES.** **a**) Ideogram of the human X chromosome, in red the region q22.1 where the *BTK* gene is located, and the BTK isoform taken from UCSC Genome Browser. **b**) Family pedigree showing the inheritance model of BTK mutation in both siblings. **c**) The box represents a zoom in the region where the mutation is located. Red and blue rectangles represent the forward and reverse NGS reads covering the variants, followed by the Sanger sequencing eletrofluorograms for the same region.

**Reference**

[1. Okonechnikov K, Golosova O, Fursov M, UGENE team. Unipro UGENE: a unified bioinformatics toolkit. Bioinformatics. 2012;28:1166–7.](http://paperpile.com/b/e5Wprt/Q44n)

[2. Langmead B, Salzberg SL. Fast gapped-read alignment with Bowtie 2 [Internet]. Nature Methods. 2012. p. 357–9. Available from:](http://paperpile.com/b/e5Wprt/wi8b) <http://dx.doi.org/10.1038/nmeth.1923>

[3. Li H, Handsaker B, Wysoker A, Fennell T, Ruan J, Homer N, et al. The Sequence Alignment/Map format and SAMtools. Bioinformatics. 2009;25:2078–9.](http://paperpile.com/b/e5Wprt/PzSY)

[4. DePristo MA, Banks E, Poplin R, Garimella KV, Maguire JR, Hartl C, et al. A framework for variation discovery and genotyping using next-generation DNA sequencing data. Nat Genet. 2011;43:491–8.](http://paperpile.com/b/e5Wprt/9KDO)

[5. Van der Auwera GA, Carneiro MO, Hartl C, Poplin R, Del Angel G, Levy-Moonshine A, et al. From FastQ data to high confidence variant calls: the Genome Analysis Toolkit best practices pipeline. Curr Protoc Bioinformatics. 2013;43:11.10.1–11.10.33.](http://paperpile.com/b/e5Wprt/W6rf)

[6. Cingolani P, Platts A, Wang LL, Coon M, Nguyen T, Wang L, et al. A program for annotating and predicting the effects of single nucleotide polymorphisms, SnpEff: SNPs in the genome of Drosophila melanogaster strain w1118; iso-2; iso-3. Fly . 2012;6:80–92.](http://paperpile.com/b/e5Wprt/6D4n)

[7. Sherry ST, Ward MH, Kholodov M, Baker J, Phan L, Smigielski EM, et al. dbSNP: the NCBI database of genetic variation. Nucleic Acids Res. 2001;29:308–11.](http://paperpile.com/b/e5Wprt/K3zs)

[8. 1000 Genomes Project Consortium, Auton A, Brooks LD, Durbin RM, Garrison EP, Kang HM, et al. A global reference for human genetic variation. Nature. 2015;526:68–74.](http://paperpile.com/b/e5Wprt/AFwp)

[9. Lek M, Karczewski KJ, Minikel EV, Samocha KE, Banks E, Fennell T, et al. Analysis of protein-coding genetic variation in 60,706 humans. Nature. 2016;536:285–91.](http://paperpile.com/b/e5Wprt/xdAU)

[10. Warren RL, Choe G, Freeman DJ, Castellarin M, Munro S, Moore R, et al. Derivation of HLA types from shotgun sequence datasets. Genome Med. 2012;4:95.](http://paperpile.com/b/e5Wprt/2Wce)
