# Supplementary figures and images for: Clinical and genetic findings in two siblings with X-Linked agammaglobulinemia and bronchiolitis obliterans: a case report
Source: BMC Pediatr. 2022 Apr 5;22:181. doi: 10.1186/s12887-022-03245-x (PMC8981605; doi:10.1186/s12887-022-03245-x)

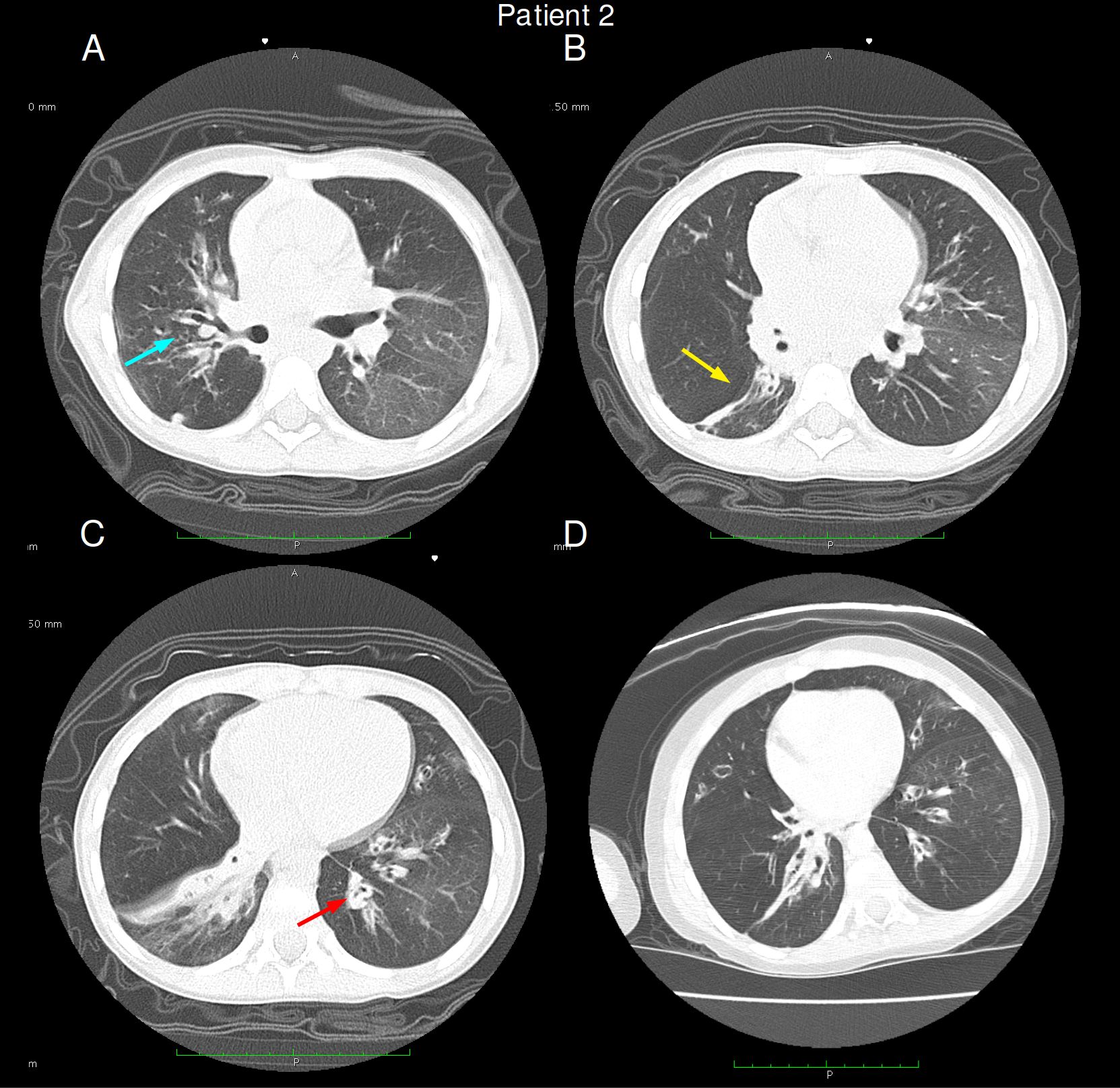

Supplement: Supplementary file 1 — Additional file 1: Table S1. Prediction of HLA alleles present in each patient. Figure S1. Chest CT scan of patient 2. a-c) was obtained in 2015 at 3y7mo, whereas d was obtained in late 2017 at 6 y.o. Cyan arrows show bronchial wall thickening. Red arrows indicate mosaic attenuation patterns and Bronchiectasis. Yellow arrow shows atelectasis. Figure S2. Genetic diagnosis of XLA patients using WES. a) Ideogram of the human X chromosome, in red the region q22.1 where the BTK gene is located, and the BTK isoform taken from UCSC Genome Browser. b) Family pedigree showing the inheritance model of BTK mutation in both siblings. c) The box represents a zoom in the region where the mutation is located. Red and blue rectangles represent the forward and reverse NGS reads covering the variants, followed by the Sanger sequencing eletrofluorograms for the same region. [file 12887_2022_3245_MOESM1_ESM.zip › Figure_S1R2.png]

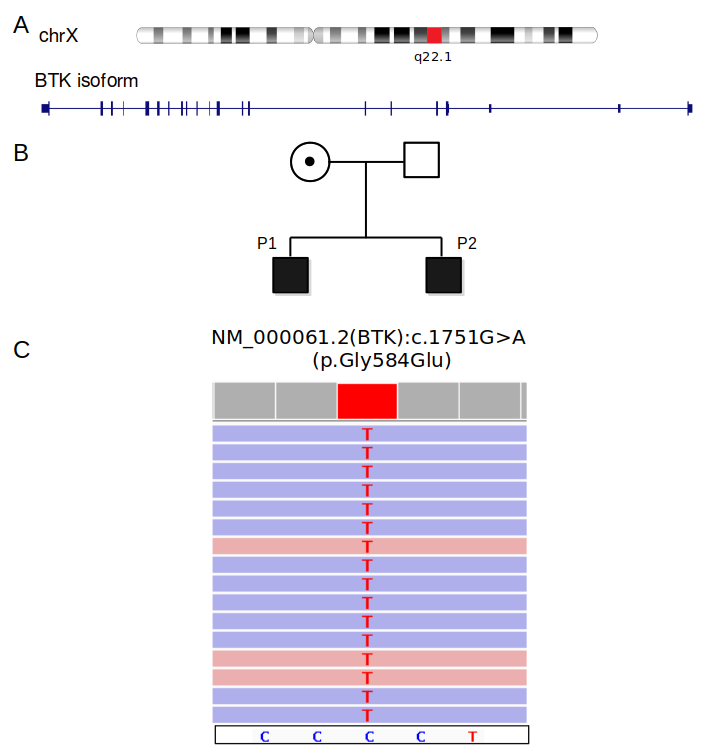

Supplement: Supplementary file 1 — Additional file 1: Table S1. Prediction of HLA alleles present in each patient. Figure S1. Chest CT scan of patient 2. a-c) was obtained in 2015 at 3y7mo, whereas d was obtained in late 2017 at 6 y.o. Cyan arrows show bronchial wall thickening. Red arrows indicate mosaic attenuation patterns and Bronchiectasis. Yellow arrow shows atelectasis. Figure S2. Genetic diagnosis of XLA patients using WES. a) Ideogram of the human X chromosome, in red the region q22.1 where the BTK gene is located, and the BTK isoform taken from UCSC Genome Browser. b) Family pedigree showing the inheritance model of BTK mutation in both siblings. c) The box represents a zoom in the region where the mutation is located. Red and blue rectangles represent the forward and reverse NGS reads covering the variants, followed by the Sanger sequencing eletrofluorograms for the same region. [file 12887_2022_3245_MOESM1_ESM.zip › Figure_S2R2.png]
